# Long- and short-term CDK5 knockdown prevents spatial memory dysfunction and tau pathology of triple transgenic Alzheimer’s mice

**DOI:** 10.3389/fnagi.2014.00243

**Published:** 2014-09-10

**Authors:** John F. Castro-Alvarez, S. Alejandro Uribe-Arias, Kenneth S. Kosik, Gloria P. Cardona-Gómez

**Affiliations:** ^1^Cellular and Molecular Neurobiology Area, Neuroscience Group of Antioquia, Faculty of Medicine, University Research Headquarters, University of AntioquiaMedellín Colombia; ^2^Department of Molecular Cellular Developmental Biology, Neuroscience Research Institute, University of California, Santa BarbaraSanta Barbara, CA USA

**Keywords:** Tau pathology, cognitive dysfunction, Alzheimer’s disease, CDK5RNAi, long-term gene therapy

## Abstract

CDK5 is a member of the cyclin-dependent kinase family with diverse functions in both the developing and mature nervous system. The inappropriate activation of CDK5 due to the proteolytic release of the activator fragment p25 from the membrane contributes to the formation of neurofibrillary tangles and chronic neurodegeneration. At 18 months of age 3xTg-AD mice were sacrificed after 1 year (long term) or 3 weeks (short term) of CDK5 knockdown. In long-term animals CDK5 knockdown prevented insoluble Tau formation in the hippocampi and prevented spatial memory impairment. In short-term animals, CDK5 knockdown showed reduction of CDK5, reversed Tau aggregation, and improved spatial memory compared to scrambled treated old 3xTg-AD mice. Neither long-term nor short-term CDK5 knock-down had an effect on old littermates. These findings further validate CDK5 as a target for Alzheimer’s disease both as a preventive measure and after the onset of symptoms.

## INTRODUCTION

Alzheimer’s disease (AD) is the most common cause of dementia in people over 65 with about 24 million people affected worldwide (Reitz et al., 2011). AD is characterized by abnormal folding and aggregation of β-amyloid protein (βA) as amyloid plaques and hyperphosphorylated Tau as neurofibrillary tangles (NFT). These proteinopathies cause brain atrophy with marked cognitive impairment as the disease progresses (Querfurth and LaFerla, 2010).

Tau is a microtubule (MT)-associated protein that promotes MT assembly and stabilizes assembled MTs. These functions are important in the growth of neurons during development and in axonal transport in mature neurons (Murphy and Borisy, 1975; Mandelkow and Mandelkow, 2012). The phosphorylation/dephosphorylation rate of Tau by different kinases and phosphatases regulates MT function (Wang et al., 2007). An abnormal increase in Tau phosphorylation is associated with loss of its function, formation of paired helical filaments (PHF) and extracellular accumulation in NFT (Grundke-Iqbal et al., 1986; Kosik et al., 1986; Wang et al., 2007).

Cyclin-dependent kinase 5 (CDK5) is one of several kinases involved in the phosphorylation of Tau (Paudel et al., 1993; Noble et al., 2003). Pharmacological inhibition and genetic knock down of CDK5 have been reported as therapeutic approaches to the tauopathies (Alvarez et al., 1999; Zheng et al., 2005, 2010; Piedrahita et al., 2010; Crews et al., 2011), but the importance of this kinase and its role in neurons requires further study.

Unlike other members of the CDK family, CDK5 is not involved directly in the cell cycle. It is critical in neurodevelopment and regulates neuronal functions, such as neuronal maturation, migration, neurotransmission and synaptic plasticity (Fu et al., 2005; Ip and Tsai, 2008). CDK5 is expressed in different tissues, but its activity is higher in neural tissue due to the presence of the activators, p35 and p39, that are found predominantly in the nervous system (Tsai et al., 1994; Zheng et al., 1998). Activation of calcium-dependent proteases m-calpain or μ-calpain can cleave p35, generating p25 protein, which induces prolonged activation (Patrick et al., 1999; Lee et al., 2000). In the setting of a brain insult, such as head injury, calcium influx occurs and p35 is cleaved near its membrane attachment site. As a result CDK5 can diffuse through the cytoplasm and phosphorylate inappropriate substrates including Tau (Cruz et al., 2003). Inappropriate localization of CDK5 can contribute to the formation of NTF by Tau hyperphosphorylation and release from MTs (Patrick et al., 1999; Cruz et al., 2003; Noble et al., 2003), thereby inducing the breakdown of axonal transport with resulting mitochondrial and lysosomal dysfunction (Wang et al., 2009; Mandelkow and Mandelkow, 2012). These events represent one plausible pathway toward the phenotype associated with AD NFTs and other tauopathies such as Progressive Supranuclear Palsy (PSP) and Frontotemporal Dementia and Parkinsonism linked to chromosome 17 (FTDP-17; Lee et al., 2011).

Previously, we designed a microRNA-based short hairpin (sh-miR) against CDK5, which reduced CDK5 protein levels *in vitro* and *in vivo*. We reported that a reduction in CDK5 levels can reverse Tau phosphorylation*in vitro* and *in vivo* in wild type (WT) mice and triple transgenic AD model (3xTg-AD; Piedrahita et al., 2010). Building on these pre-clinical studies and to establish the therapeutic efficacy of CDK5 silencing in 3xTg-AD mice, we determined suitable delivery times to obtain reproducible and beneficial effects of shCDK5miR therapy using Adeno-Associated Virus (AAV) vectors. At 1 year and at 3 weeks after 3xTg-AD mice were injected in CA1, learning and spatial memory was tested in the Morris Water Maze (MWM). We found improved performance at both time points and reversal of Tau hyperphosphorylation and insoluble tau.

## MATERIALS AND METHODS

### RNAi DESIGN

RNAi sh-miR sequences for silencing of CDK5 (shCDK5miR) and scrambled RNA sequence as control (shSCRmiR) were based on previously published sequences (Chang et al., 2006; Piedrahita et al., 2010). These sequences were cloned into human miR-30-based stem loops by polymerase extension of overlapping DNA oligonucleotides. For cloning of RNAi vectors for AAV production, the following primers were used for polymerase extension: shCDK5miR, forward primer 5′-AAAACTCGAGTGAGCGCTGACCAAGCTGCCAGACTATACT-GTAAAGCCACAGATGGG-3′, and shCDK5miR, reverse primer, 5′-AAAAACTAGTAGGCGTTGACCAAGCTGCCAGACTATAC-CCATCTGTGGCTTTACAG-3′, or shSCRmiR, forward primer, 5′-AAAACTCGAGTGAGCGCA-CCATCGAACCGTCAGAGTT-ACTGTAAAGCCACAGATGGG-3′, and shSCRmiR reverse primer, 5′-AAAAACTAGTAGGCGTACCATCGAACCGTCAGA-GTTACCC-ATCTGTGGCTTTACAG-3′. These extension products were digested with XhoI and SpeI for directional cloning into a U6 expression plasmid cut with XhoI and XbaI (Boudreau et al., 2008).

### VIRAL PARTICLE PRODUCTION

The protocol to produce AAV particles utilized large-scale production of heterologous proteins by Sf9 insect cells culture for co-infecting recombinant baculovirus derived from the *Autographa californica* nuclear polyhedrosis virus (Urabe et al., 2002). shCDK5miR and shSCRmiR expression cassettes, driven by the mouse U6 promoter, were cloned into pAAV.CMV.humanized Renilla GFP (hrGFP), which contains AAV serotype 2/5 inverted terminal repeats, and a CMV-hrGFP-simian virus 40 poly(A) reporter cassette (Urabe et al., 2002; Boudreau et al., 2009). AAV titers were determined by using quantitative PCR and/or DNA slot blot analysis. The AAV were dialyzed before use.

### ANIMAL PROCEDURES

A total of 36 6-month-old-3xTg-AD, 20 6-month-old-control mice, 34 18-month-old-3xTg-AD and 20 18-month-old-control mice (Oddo et al., 2003) from our in-house, specific pathogen-free (SPF) colony at the vivarium at SIU, University of Antioquia, Medellín, Colombia, were kept on a 12:12-h dark:light cycle and received food and water *ad libitum*. Animals were handled according to Colombian standards (law 84/1989 and resolution 8430/1993) and the NIH animal welfare care guidelines (Public Law 99-158, November 20, 1985, “Animals in Research”). Special care was taken to minimize animal suffering and to reduce the number of animals used.

Animals were anesthetized [ketamine (5%) and xylazine (2%) to 50:5 dosage mg/kg] and bilaterally injected with 1 μL of AAV2-shSCRmiR (shSCRmiR) or AAV2-shCDK5miR (shCDK5miR) into both hippocampi (bregma coordinates were –1.7 anteroposterior, 0.8 (right) and –0.8 (left) lateral, and 1.75 mm dorsoventral). Injections were performed with a 10 μL Hamilton syringe at a rate of 0.1 μL/min and 5 min elapsed after infusion before withdrawal of the syringe. Experimental groups were: long-term control mice (control mice at 6 months old were injected and evaluated 1 year after injection), aged control mice (control mice at 18 months old were injected and evaluated 3 weeks later), aged 3xTg-AD mice treated for a short term (ST-3xTg-AD; mice at 18 months old were evaluated 3 weeks after injection) and 3xTg-AD mice treated for a long term (LT-3xTg-AD; mice at 6 months were evaluated 1 year after injection).

### MORRIS WATER MAZE TEST

A white plastic tank consisting of a 1 m diameter and 30 cm height was filled with water (22 ± 2°C) to a depth of 20 cm. The platform (7 cm diameter) was 1.5 cm below the surface of the water during spatial learning and 1.5 cm above the surface of the water during the visible session. Extramaze visual cues around the room remained in a fixed position throughout the experiment. Each session consisted of four successive trials (30 s inter-trial interval) and each trial began with the mice placed pseudo-randomly in one of four starting locations. Animals had been trained to remain 30 s on the platform before the initial trial began. Latency to reach the platform was evaluated with a visible platform to control any difference between experimental groups in visual-motor abilities or motivation. Six to eight hidden training sessions (four trials per session) were conducted with long-term control, aged control, ST-3xTg-AD and LT-3xTg-AD mice injected with the shSCRmiR or shCDK5miR treatment to examine spatial learning performance. If a mouse did not find the platform after a maximum of 60 s it was gently guided to the platform. Animals were then given 48 h of retention time and were tested in a probe trial of spatial reference memory for 60 s without the platform. Latency in reaching the exact platform location and time spent (s) in quadrants were recorded during the probe trial. An automated system (Viewpoint, Lyon, France) recorded the mice’s behavior.

### WESTERN BLOTTING

After behavior testing, the animals were sacrificed and the hippocampus were dissected, immediately frozen in liquid nitrogen and stored at –80°C before use. Samples were lysed on 10 mM Tris, pH 7.4; 100 mM NaCl, 1 mM EDTA, 1 mM EGTA, 10% glycerol, 1% NP40, 1 nM orthovanadate, 5 mM NaF, 1 mM phenylmethylsulfonyl fluoride and protease inhibitors cocktail (Sigma-Aldrich; Cardona-Gómez et al., 2004). Proteins were loaded on 8%, 10%, and 15% Tricine Gel and transferred to nitrocellulose membranes (GE Healthcare) at 250 mA for 2 h using an electrophoretic transfer system. The membranes were incubated overnight at 4°C with anti-CDK5 (C-8), anti-p35/p25 (Santa Cruz Biotechnology). PHF-1 monoclonal antibody, which recognizes Tau pSer-396/404 donated by P. Davies (Feinstein Institute for Medical Research, Manhasset, NY, USA), mouse AT8 (pSer202/pThr205), AT100 (pSer212/pThr214), AT180 (pThr231; Pierce Biotechnology), total Tau (Tau5; Invitrogen), and mouse anti-βIII tubulin (Promega). IRDye 800CW goat anti-mouse or rabbit (LI-COR) and anti-mouse IgG or anti-rabbit IgG peroxidase conjugated (Pierce Biotechnology), were used as secondary probes. The blots were developed using the Odyssey Infrared Imaging System or chemiluminescence method (ECL Western blotting system; GE Healthcare) followed by an exposure to a radiographic film (ECL Hyperfilm; GE Healthcare). The films were analyzed using Quantity One, version 4.3.0 (Bio-Rad).

### SOLUBLE AND SARKOSYL-INSOLUBLE Tau

Preparations of Sarkosyl insoluble Tau were previously described (Greenberg and Davies, 1990; DeTure et al., 2002). Briefly, hippocampi from ST-3xTg-AD and LT-3xTg-AD mice were lysed on 10 mM Tris, pH 7.4; 100 mM NaCl, 1 mM EDTA, 1 mM EGTA, 10% glycerol, 1% NP40, 1 nM orthovanadate, 5 mM NaF, 1 mM phenylmethylsulfonyl fluoride, and protease inhibitors cocktail (Sigma-Aldrich). Lysates were spun at 13,000 rpm, 4°C, 10 min, a fraction of the supernatant was stored as the soluble fraction. Remaining fraction was diluted in Sarkosyl buffer (50 mM Tris–HCl, pH 7.4; 0.15 M NaCl, 1% lauryl sarcosine, protease inhibitor cocktail) and spin at 13,000 rpm, 4°C, 10 min. The supernatant was incubated for 30 min/RT and centrifuged at 40,000 rpm/2 h. The pellet was diluted in Sarkosyl buffer and stored as insoluble fraction. The soluble and insoluble fractions were analyzed with PHF1 and β-actin (Sigma-Aldrich) monoclonal antibodies by western blot, as described above.

### CDK5 KINASE ASSAY

Hippocampi from ST-3xTg-AD, LT-3xTg-AD, and long-term control mice were dissected and rapidly frozen in liquid nitrogen immersion. Brain tissue was thawed on ice and homogenized, incubated for 15 min on ice, and centrifuged at 13,000 rpm/4°C. CDK5 was immunoprecipitated with polyclonal anti-CDK5 (C-8) antibody. After 24 h of incubation, protein G-Sepharose beads were resuspended in 200 μL of kinase assay buffer (20 mM Tris–HCl, pH 7.5, 100 μM sodium orthovanadate, 10 mM MgCl_2_, 50 mM NaCl, 1 mM DTT, and 1 mM NaF), and ATP was added to the resuspended beads at a 10-fold excess (0.5 mM). Histone from calf thymus type III-S (Sigma-Aldrich) was added as a substrate for CDK5 at a final concentration of 6 μM, and then the reaction was gently vortexed and incubated at 37°C for 30 min. Reaction were stopped with 5 μL of loading buffer (250 mM Tris–HCl, 10% SDS, 30% glycerol, 0.5 M DTT, 0.02% bromophenol blue), immediately followed by 5 min incubation at 95°C. Western blotting detection for anti-histone H1 phosphorylated, CDK5, and IgG were made as described above.

### IMMUNOFLUORESCENCE

Mouse brains were fixed with 4% paraformaldehyde in PBS, cryopreserved with 30% sucrose and stored at 20°C. Brains were cut in 50 μm coronal sections with a vibratome (Leica 1000) and treated with 50 mM NH_4_Cl for 10 min at room temperature. Slices were preincubated 1 h in 1% BSA with 0.3% Triton X-100 in 0.1 M PB. Primary antibodies were incubated overnight at 4°C. We used PHF-1 monoclonal antibody, which recognizes Tau phospho-Ser-396/404. Secondary antibodies were conjugated to fluorophore Alexa 594 (Molecular Probes). The slices were observed by Confocal-DSU microscope Olympus IX-8 and analyzed as individual images for PHF-1 expression and GFP as a reporter gene. Deconvolution, maximal projection and fluorescence intensity were done using Image Scope-Pro software (Media Cybernetics) and Cell software (Olympus), respectively.

### STATISTICS

At least *n* = 3–4 mice were used for histological and biochemical studies from *n* = 10–18 animals used for behavioral evaluation. Parametric data was compared using Student’s *t*-test for contrast groups. Nonparametric data was evaluated by the Mann–Whitney *U* test. Escape latency during the visible and hidden training was determined by repeated-measures ANOVA with session as a within-subject factor and treatment (shSCRmiR, shCDK5miR) as between-subjects factor. In the hidden trials separately and probe trial used Student’s *t*-test for latency and two-way ANOVA with treatment-time in quadrants as between subject factors. SPSS was used and results were considered significant when *p* ≤ 0.05. Values were expressed as mean ± SEM.

## RESULTS

### LONG STANDING PREVENTION OF SPATIAL MEMORY IMPAIRMENT IN 3xTg-AD 1 YEAR AFTER CDK5 KNOCKDOWN

**FIGURE 1:**
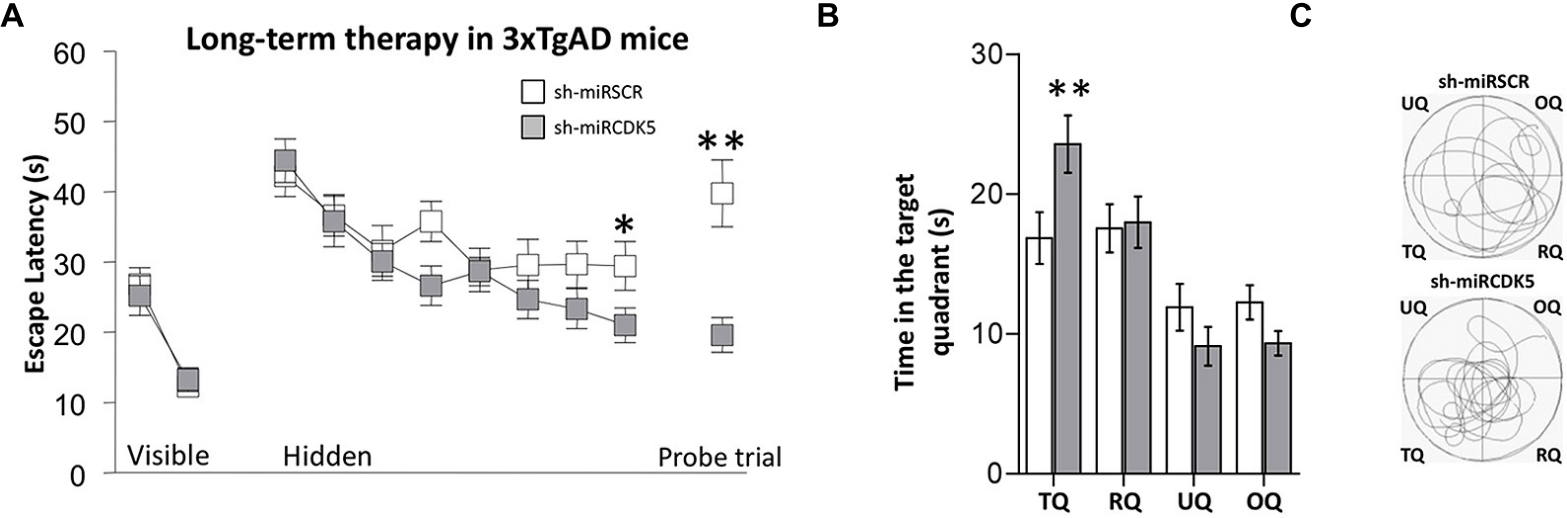
**Long-term therapy (1 year after injection at 6 months of age) with shCDK5miR in 3xTg-AD mice prevented the spatial memory deficit.** Therapy with shCDK5miR had a beneficial effect on spatial memory by MWM testing. **(A)** Values of latency to reach the hidden platform during training show significant difference in the learning and probe trial tests at 48 h. This effect was replicated in **(B)** time spent in the target quadrant and **(C)** path plots for the median path length. (TQ = target quadrant, RQ = Right quadrant, UQ = upper quadrant and OQ = oppositive quadrant.) Data are presented as mean ± SEM. *n* = 18 by each group, *p < 0.05, **p < 0.01.

Six-month-old 3xTg-AD mice were injected with either shCDK5miR or shSCRmiR in CA1 and were evaluated by MWM test 1 year post-injection. Before cognitive testing all the animals found the visible platform quickly indicating no visual, motor or motivational deficits [session effect *F*(1,34) = 53.245; *p* < 0.001; treatment effect *F*(1,34) = 0.012; *p* = 0.915; session-treatment effect *F*(1,34) = 0.5; *p* = 0.484; **Figure 1A**, Visible platform]. In the learning test, shSCRmiR treated mice did not improve during hidden platform training with respect to shCDK5miR treated mice [session effect *F*(7,238) = 11.04; *p* < 0.001; treatment effect *F*(1,34) = 1.848; *p* = 0.183; session-treatment effect *F*(7,224) = 1.316; *p* = 0.243; **Figure 1A**, Hidden platform]. The performance of shCDK5miR treated mice improved in the last learning session [session 8 (Mann–Whitney *U* test; *p* = 0.038] compared to the shSCRmiR-injected mice, and thus the shCDK5miR treated mice achieved a performance similar to long-term control mice (**Figure 3A**).

In the probe trial or retention test, the 3xTg-AD mice injected with shCDK5miR showed a significant reduction in the latency to reach the platform position (Mann–Whitney *U* test; *p* = 0.004; **Figure 1A**, Probe trial). These results were corroborated by an analysis of the time spent in quadrants by tracking motion. shCDK5miR-treated animals showed a significantly increased time spent in searching the platform in the target quadrant (TQ) with respect to the other quadrants [two-way ANOVA *F*(1,34) = 5.948; *p* = 0.02; **Figure 1B**] and an increase in the path length in the platform quadrant (**Figure 1C**). Thus, CDK5 knockdown can contribute to prevention of spatial learning impairment in aged 3xTg-AD mice.

Control mice from the same line (PS1_M146V_ knock-in) at 6 months of age were injected in CA1 with AAV containing shCDK5miR or shSCRmiR. All mice were evaluated by the MWM test 1 year post-injection. Performance with the visible platform demonstrated no deficits in visual, motor or motivational functions in the injected animals [session effect *F*(1,16) = 26.247; *p* < 0.001; treatment effect *F*(1,16) = 0.036; *p* = 0.853; session-treatment effect *F*(1,16) = 0.305; *p* = 0.589; **Figure 3A**, Visible platform]. Both shCDK5miR and shSCRmiR groups could learn the tasks in the hidden platform training [session effect *F*(7,105) = 12.395; *p* < 0.001], and there were no differences searching the location of the hidden platform [treatment effect *F*(1,15) = 1.901; *p* = 0.188; session-treatment effect *F*(7,105) = 0.799; *p* = 0.606; **Figure 3A**, Hidden platform]. 48 h after the last learning trial was assessed a probe trial (without platform), did not reveal any differences in the latency in reaching the platform-demarcated area (*t*(15) = 1.46; *p* = 0.165) or time spent in the target quadrant [two-way ANOVA *F*(3,64) = 8.852; *p* = 0.588; **Figure 3A** (Probe trial), and **Figures 3B,C**].

### REDUCED INSOLUBLE Tau AND HYPERPHOSPHORYLATED Tau IN THE HIPPOCAMPI OF 3xTg-AD FOLLOWING LONG-TERM CDK5 KNOCKDOWN

**FIGURE 2:**
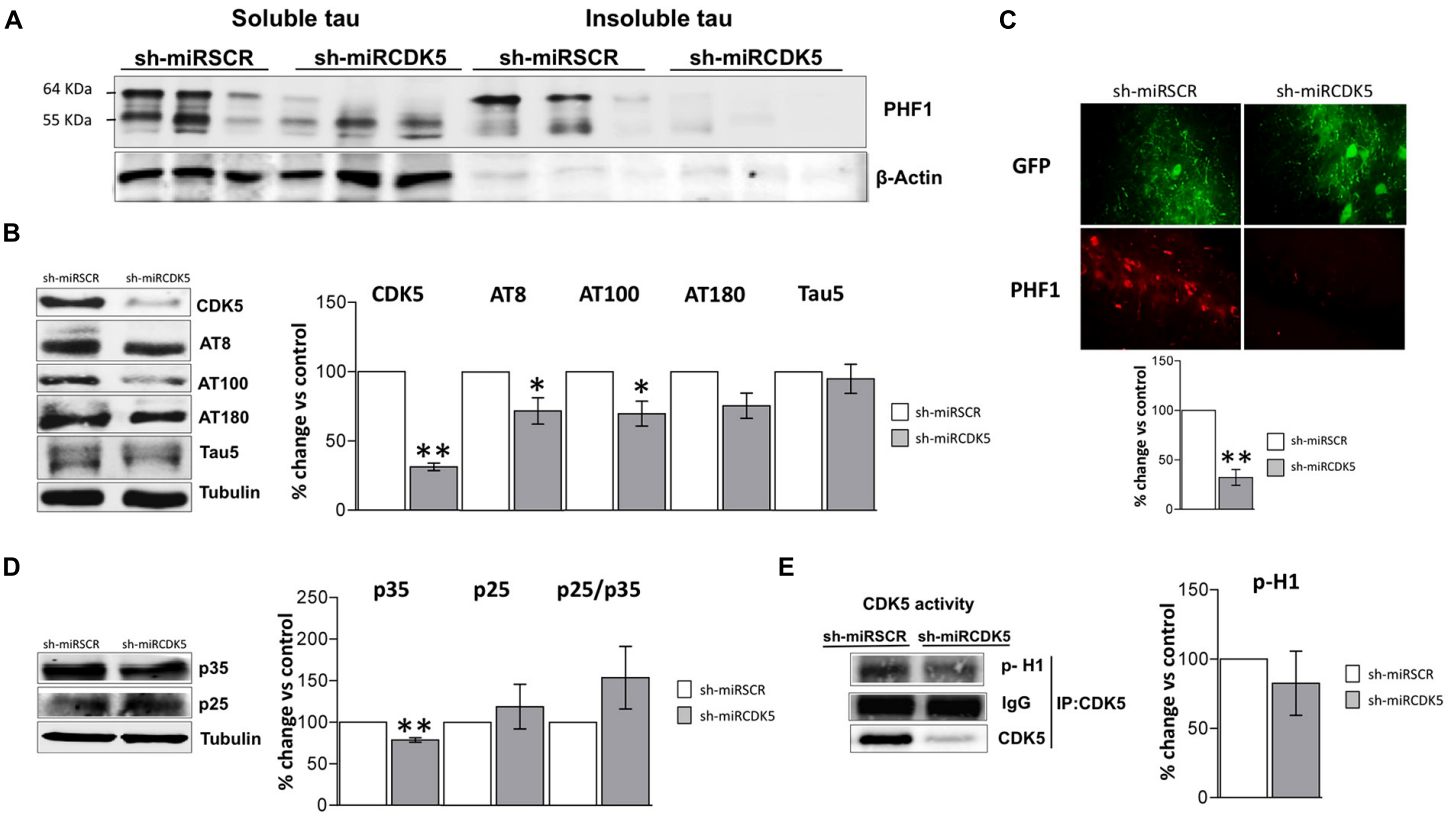
**Long-term therapy in 3xTg-AD mice with shCDK5miR can prevent accumulation of insoluble and hyperphosphorylated Tau.** Long-term therapy prevented insoluble Tau accumulation (64 kDa band) as measured by **(A)** sarkosyl-insoluble and soluble Tau evaluated with PHF1 antibody, β-actin was used as loading control. **(B)** Likewise, long-term shCDK5miR maintained decreased CDK5 protein levels as well as reduced AT8 and AT100 in hippocampal lysates of 3xTg-AD mice. Representative blots are shown. Tubulin was used as loading control. Densitometry quantification was done. *n* = 3, *p < 0.05, **p < 0.01. **(C)** PHF1 fluorescence intensity corroborated that Tau hyperphosphorylation diminished with long-term therapy of shCDK5miR in CA1 of 3xTg-AD mice. PHF1 in red (Alexa 594) and GFP in green. Magnification 60×. Scale bar, 50 μm. *n* = 3. *p < 0.05, **p < 0.01. **(D)** p35 levels remain reduced 1 year after treatment with shCDK5miR. p25 and p25/p35 ratio was slightly increased but did not achieve statistical significance. Representative blots are shown. Tubulin was used as loading control. Densitometry quantification was done. *n* = 3, *p < 0.05, **p < 0.01. **(E)** CDK5 kinase activity evaluated by histone phosphorylation in long-term treatment in 3xTgAD mice. A band corresponding to the IgG heavy chain was detected and used as loading control. Densitometry quantification was done. *n* = 3, *p < 0.05, **p < 0.01.

To detect a biochemical effect of CDK5 knockdown on hyperphosphorylation of Tau, the Tau soluble and sarkosyl-insoluble fraction were separated in LT-3xTg-AD hippocampi. 6-month-old 3xTg-AD animals injected with shSCRmiR and sacrificed 1 year later showed a PHF1 immunoreactive band in 65 kDa (pSer-396/404; Greenberg and Davies, 1990; Kimura et al., 2010), characteristic of hyperphosphorylated Tau in the soluble fraction and in sarkosyl-insoluble fraction (**Figure 2A**). This band was not present in shCDK5miR treated animals in both the sarkosyl-insoluble fraction and in the soluble fraction (**Figure 2A**).

CDK5 reduction was verified after 1 year of treatment in the shCDK5miR treated mice. CDK5 protein levels were significantly reduced (31.3% ± 2.7 vs control; *p* = 0.001; **Figure 2B**). Several phosphorylation sites in total lysates were evaluated after CDK5 reduction. There was a significant decrease in the AT8 (pSer202/pThr205; 71.9 ± 9.5% vs control; *p* = 0.049) and AT100 (pSer212/pThr214; 69.7 ± 9% vs control; *p* = 0.039) immunoreactivities, but AT180 (pThr231) did not achieve statistical significance (75.4 ± 9.1%; *p* = 0.114; **Figure 2B**). Total Tau blotted with Tau5 did not differ between the groups (95 ± 10.5% vs control; *p* = 0.34; **Figure 2B**). PHF1 reactivity was quantified by immunofluorescence of CA1 that showed reduced signal in the severity of Tau pathology in the shCDK5miR group (32.2 ± 8% vs control; *p* = 0.001; **Figure 2C**). Taken together the most prominent effect of CDK5 knockdown is on PHF1 hyperphosphorylated Tau in the sarkosyl insoluble fraction.

CDK5 activity and its effect on tauopathy can be associated with the modulation of its activator p35 and p25 cleaved form. Thus, we evaluated p35/p25 levels and CDK5 activity in our samples. p35 (78.5 ± 2.7% vs control; *p* = 0.004) was significantly decreased, but there was no change in p25 (118.9 ± 26.9% vs control; *p* = 0.533) after 1 year of treatment with shCDK5miR in 3xTg AD mice and there was no significant change in the ratio p25/p35 (153.8 ± 37.6% vs control; *p* = 0.2476; **Figure 2D**). Also, the capacity of CDK5 to phosphorylate an *in vitro* substrate such as histone H1 in 3xTg-AD knockdown mice was not changed after 1 year (82.6 ± 23.2% vs control; *p* = 0.531; **Figure 2E**).

**FIGURE 3:**
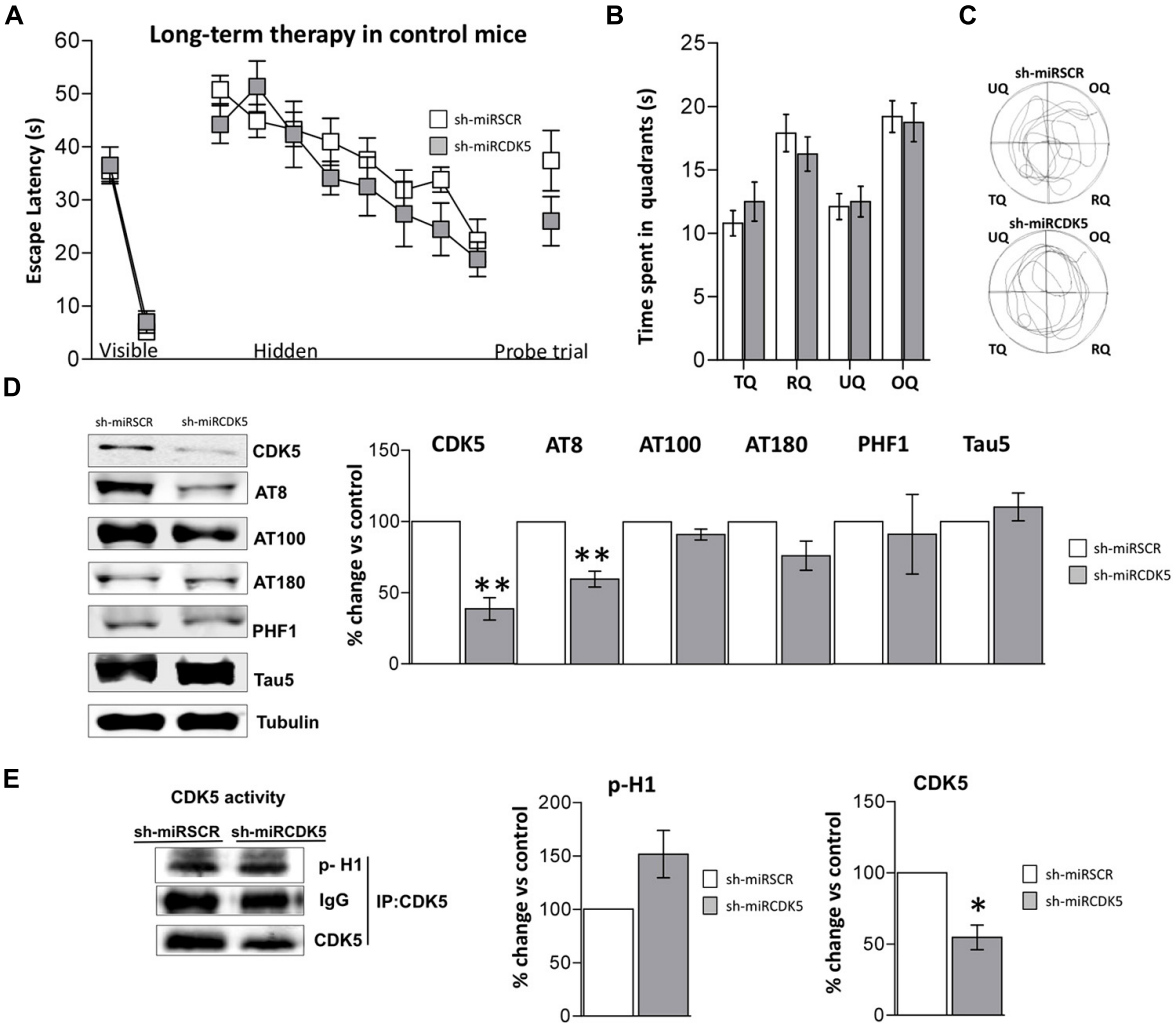
**Long-term treatment with CDK5 RNAi’s reduced phosphorylated Tau in control animals (littermates with only the PS1_**M146V**_knock-in) but did not affect spatial memory.** Morris Water Maze (MWM) testing was performed in 18-month-old control mice 1 year after injection with shSCRmiR (white squares) and shCDK5miR (gray squares). **(A)** Values of latency to reach the visible and hidden platform during training and probe trial tests 48 h after training. **(B)** Time spent in the target quadrant and **(C)** path plots for the median path length during the probe trial. (TQ = target quadrant, RQ = right quadrant, UQ = upper quadrant and OQ = oppositive quadrant.) Data are presented as mean ± SEM. *n* = 10 by each group. *p < 0.05, **p < 0.01. **(D)** Steady-state levels of CDK5 and AT8 decreased after injection of shCDK5miR in hippocampal lysates of aged littermate mice injected with shSCRmiR (white bars) and shCDK5miR (gray bars). There was no statistical significance between shSCRmiR and shCDK5miR in AT100, AT180, PHF1 and Tau5 levels. Representative blots are shown. Tubulin was used as loading control. Densitometry quantification was done, RU = relative units. *n* = 4. *p < 0.05, **p < 0.01. **(E)** CDK5 kinase activity evaluated by histone phosphorylation in long-term treatment in control mice. A band corresponding to the IgG heavy chain was detected and used as loading control. Densitometry quantification was done. *n* = 3, *p < 0.05, **p < 0.01.

Control mice at 6 months of age injected in CA1 with AAV containing shCDK5miR or shSCRmiR. One year later, after MWM testing, animals were sacrificed and CDK5 protein levels in hippocampal lysates corroborated the shCDK5miR-induced reduction. CDK5 was diminished in the hippocampi of aged control shCDK5miR-treated animals in comparison to shSCRmiR-treated animals (38.7 ± 7.9% vs control; *p* = 0.004; **Figure 3D**). We immunoblotted Tau to evaluate its phosphorylation state at the AT8, AT100, AT180, and PHF-1 epitopes. CDK5 silencing reduced Tau phosphorylation at the AT8 (59.8 ± 5.6% vs control; *p* = 0.005), with tendency toward a decrease of AT180 (76.2 ± 23.7% vs control; *p* = 0.103); but not at the PHF1 (91.1 ± 27.9% vs control; *p* = 0.771), AT100 (91.1 ± 3.8% vs control; *p* = 0.101) sites or total Tau (110.2 ± 9.7% vs control; *p* = 0.37) with Tau5 antibody in long-term control mice (**Figure 3D**). In the long-term control mice, capacity of CDK5 to phosphorylate an *in vitro* substrate such as histone H1 has a tendency to increase, but it was not significant (151.6 ± 19.2% vs control; *p* = 0.0726; **Figure 3E**).

### SHORT-TERM EFFECTS OF CDK5 KNOCKDOWN – 3 WEEKS AFTER DELIVERY

**FIGURE 4:**
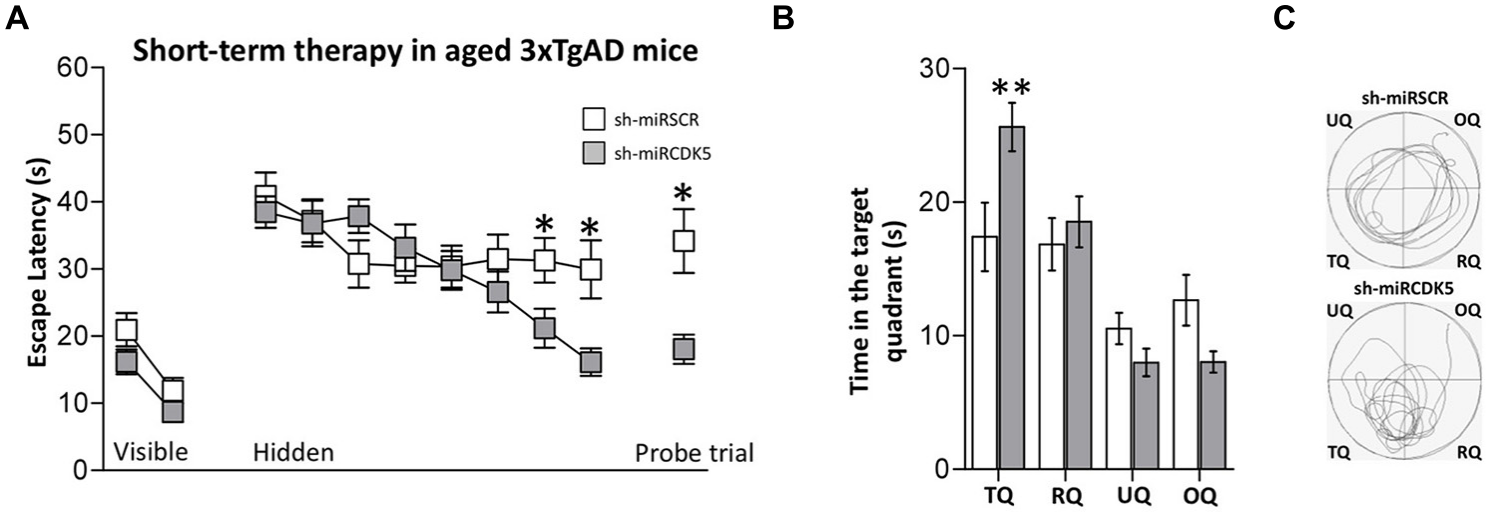
**Short-term treatment with shCDK5miR induced recovery of spatial memory.** Deficits in spatial learning were reduced in 3xTgAD mice injected with shCDK5miR (gray square) in comparison with shSCRmiR (white squares). **(A)** Values of latency to reach the visible and hidden platform during training, and probe trial tests 48 h after training. shCDK5miR injection improved the performance of 3xTgAD mice during the training with the hidden platform and the probe trial. **(B)** Time spent in the target quadrant and **(C)** path plots for the median path length that evidenced the recovery in the preference by the platform position. (TQ = target quadrant, RQ = right quadrant, UQ = upper quadrant and OQ = oppositive quadrant.) Data are presented as mean ± SEM. *n* = 17 by each group. *p < 0.05, **p < 0.01.

Animals were injected at 18 months of age and evaluated 3 weeks later. Thus the animals in this experiment were sacrificed at the same age as the long-term (1 year) treated animals described above that were treated with shCDK5miR at 6 months. These “short term” animals did not show alterations in the visible test [session effect *F*(1,32) = 25.562; *p* < 0.001; treatment effect *F*(1,32) = 3.246; *p* = 0.081; session-treatment effect *F*(1,32) = 0.218; *p* = 0.644; **Figure 4A**, Visible platform]. Although the shCDK5miR group improved their performance in the last hidden platform learning sessions [session 7 (Mann–Whitney *U* test; *p* = 0.036] and session 8 [Mann–Whitney *U* test; *p* = 0.017)], there was no session-treatment effect [session effect *F*(7,224) = 7.562; *p* < 0.001; treatment effect *F*(1,32) = 1.291; *p* = 0.264; session-treatment effect *F*(7,224) = 2.862; *p* = 0.007; **Figure 4A**, Hidden platform]. Spatial memory analysis showed a clear difference in the probe trial of the shCDK5miR group compared to the shSCRmiR animals. This result was comparable to the results in the LT-3xTg-AD performance. The latency to finding the platform in the shCDK5miR group was lower than in the shSCRmiR group (Mann–Whitney *U* test *p* = 0.015; **Figure 4A**, Probe trial) and preference by the platform quadrant was also superior in the shCDK5miR treated mice as shown by the time spent in the platform quadrant and path length [two-way ANOVA *F*(1,32) = 6.863; *p* = 0.013; **Figures 4B,C**]. These findings suggest a short-term ameliorative effect on spatial memory.

**FIGURE 5:**
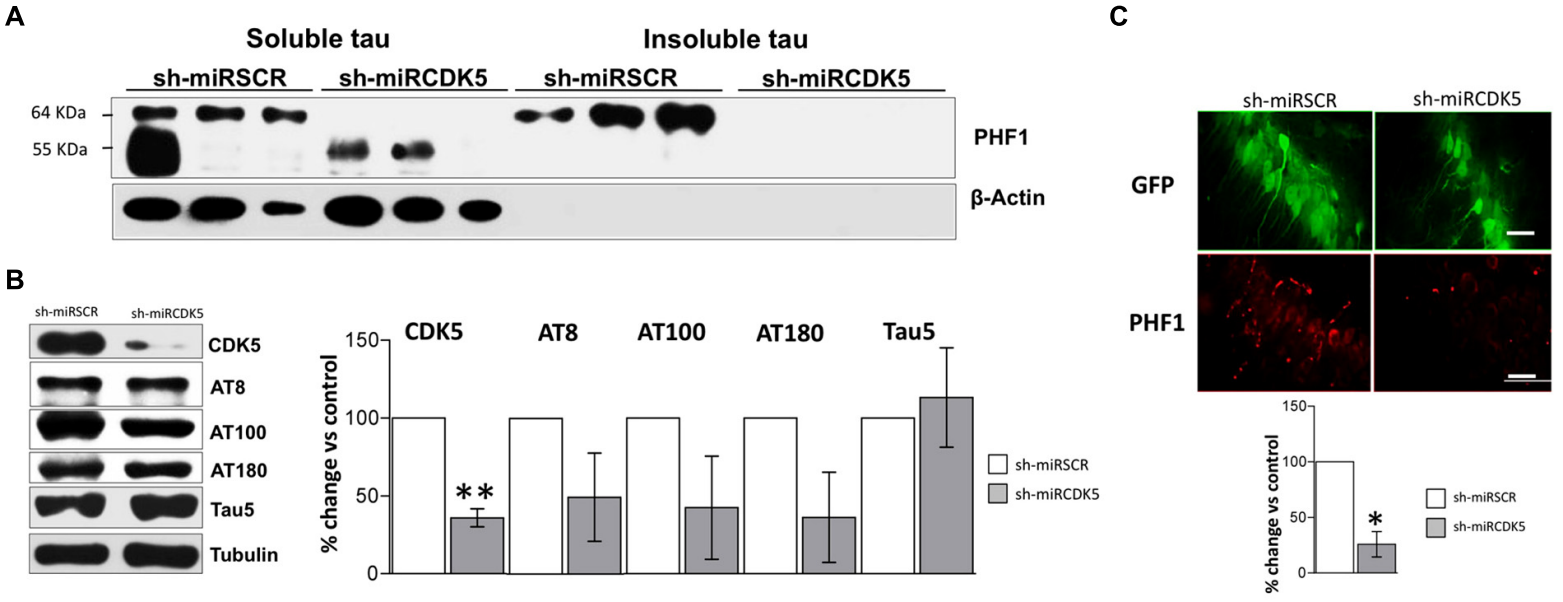
**CDK5 knockdown diminished aggregation of insoluble Tau in old 3xTg-AD mice. (A)** Separation of sarkosyl-insoluble and soluble Tau showed that shCDK5miR can decrease insoluble Tau evaluated with PHF1 antibody (64 kDa band) in a short-term treatment in 3xTgAD mice compared to control. β-actin was used as loading control. **(B)** CDK5 protein levels reduced in the CDK5 knockdown in 3xTgAD mice. AT8, AT100 and AT180 levels after injection of shCDK5miR did show any statistically significant decrease. Representative blots are shown. Tubulin was used as loading control. Densitometry quantification was done. *n* = 3, *p < 0.05, **p < 0.01. **(C)** Quantification of PHF1 evaluated by immunofluorescence in CA1 revealed that shCDK5miR decreased the intensity of fluorescence in 3xTg-AD mice. PHF1 in red (Alexa 594) and GFP in green. Magnification 60×. Scale bar, 50 μm; *n* = 3; *p < 0.05, **p < 0.01. Densitometry quantification was done. *n* = 3, *p < 0.05, **p < 0.01.

Tauopathy was significantly diminished in the ST-3xTg-AD with shCDK5miR injection. Sarkosyl-insoluble and soluble fractions showed a reduction in hyperphosphorylated Tau after 3 weeks of treatment (**Figure 5A**). We confirmed the reduction of CDK5 protein levels 3 weeks after treatment (38.8 ± 12.2% vs control; *p* = 0.006) and wide variation in AT8 (49.4 ± 28.3% vs control; *p* = 0.108) and AT100 (42.5 ± 33.14% vs control; *p* = 0.112) immunoreactivities that failed to achieve statistical significance (**Figure 5B**). AT180 did not achieve statistical significance, but did show a decreasing trend (36.2 ± 29% vs control; *p* = 0.079; **Figure 5B**). As seen in both aged control mice and ST-3xTg-AD, total Tau with Tau5 antibody did not differ between the groups (113.2 ± 31.9% vs control; *p* = 0.359; **Figure 5B**). PHF1 immunofluorescence was significantly reduced in CA1 [25.8 ± 11.39% (shCDK5miR) vs control; *p* = 0.02; **Figure 5C**]. These studies support a short-term ameliorative effect on Tau phosphorylation.

**FIGURE 6:**
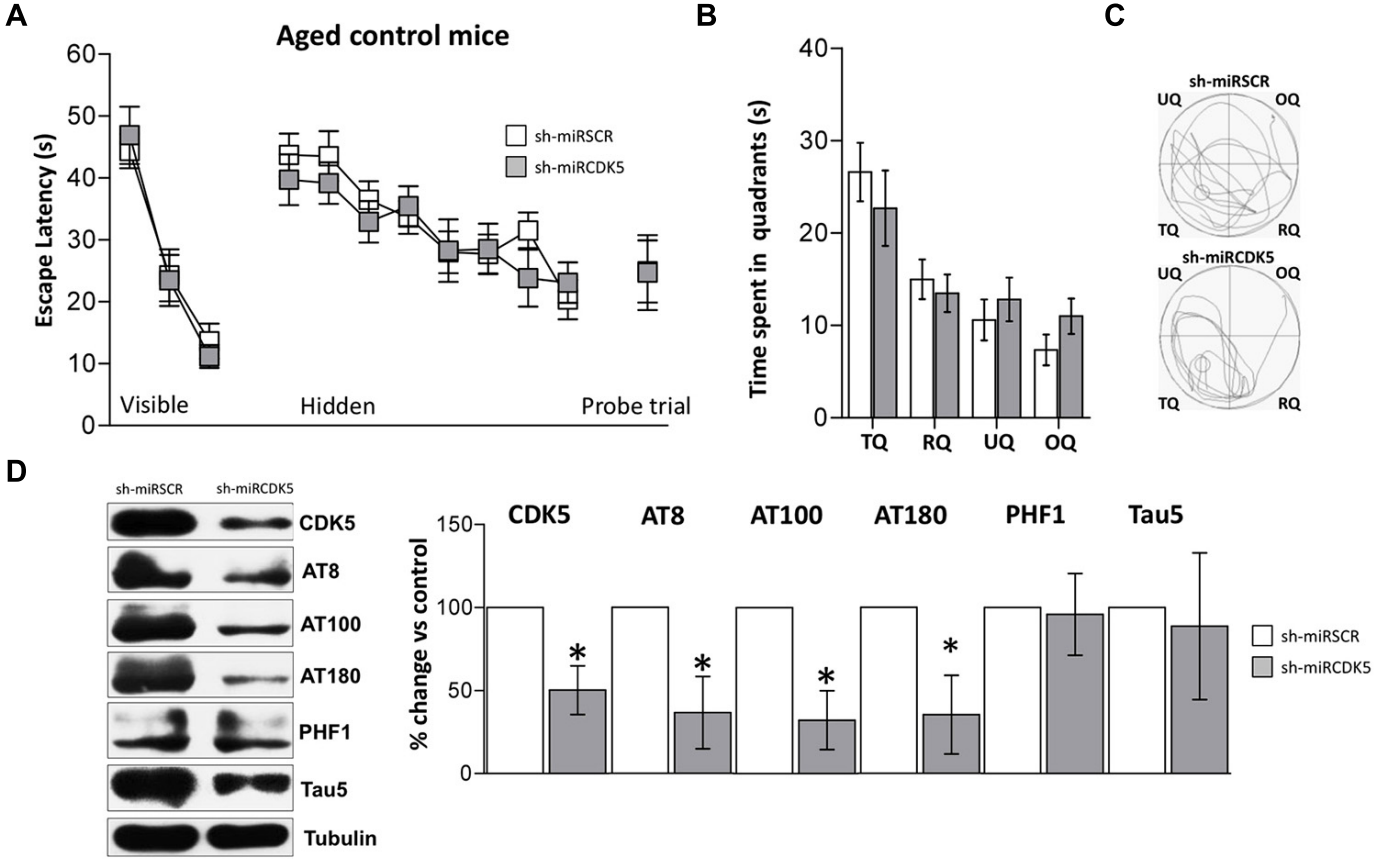
**CDK5 RNAi’s reduced phosphorylated Tau in control animals (littermates with only the PS1_M146V_knock-in) but did not affect spatial memory.** Morris Water Maze (MWM) testing was performed in 18-month-old control mice injected with shSCRmiR (white squares) and shCDK5miR (gray squares). **(A)** Values of latency to reach the visible and hidden platform during training and probe trial tests 48 h after training. **(B)** Time spent in the target quadrant and **(C)** path plots for the median path length during the probe trial. (TQ = target quadrant, RQ = right quadrant, UQ = upper quadrant and OQ = opposite quadrant.) Data are presented as mean ± SEM. *n* = 10 by each group. *p < 0.05. **(D)** Steady-state levels of CDK5, AT8 and AT100 decreased after injection of shCDK5miR in hippocampal lysates of aged littermate mice injected with shSCRmiR (white bars) and shCDK5miR (gray bars). There was no statistically significant difference between shSCRmiR and shCDK5miR in AT180, PHF1 and Tau5 levels. Representative blots are shown. Tubulin was used as a loading control. Densitometry quantification was done. RU = relative units. *n* = 4. *p < 0.05.

Control mice (PS1_M146V_knock-in) at 18 months of age were injected in CA1 with AAV containing shCDK5miR or shSCRmiR. All mice were evaluated by the MWM test 3 weeks post-injection. Performance with the visible platform demonstrated no deficits in visual, motor or motivational functions in the injected animals [session effect *F*(2,40) = 53.747; *p* < 0.001; treatment effect *F*(1,20) = 0.003; *p* = 0.958; session-treatment effect *F*(2,40) = 0.285; *p* = 0.753; **Figure 6A**, Visible platform]. Both shCDK5miR and shSCRmiR groups learned the tasks in the hidden platform training [session effect *F*(7,140) = 11.85; *p* < 0.001], and there were no differences in searching the location of the hidden platform [treatment effect *F*(1,20) = 0.262; *p* = 0.614; session-treatment effect *F*(7,140) = 0.768; *p* = 0.615; **Figure 6A**, Hidden platform]. 48 h after the last learning trial was assessed a probe trial (without platform), did not reveal any differences in the latency in reaching the platform-demarcated area [*t*(20) = 0.23; *p* = 0.982] or time spent in the target quadrant [two-way ANOVA *F*(1,20) = 0.573; *p* = 0.458; **Figure 6A** (Probe trial), and **Figures 6B,C**].

CDK5 was diminished in the hippocampi of aged control shCDK5miR-treated animals in comparison to shSCRmiR-treated animals (50.2 ± 14.6% vs control; *p* = 0.021; **Figure 6D**). We immunoblotted Tau to evaluate its phosphorylation state at the AT8, AT100, AT180, and PHF-1 epitopes. CDK5 silencing reduced Tau phosphorylation at the AT8 (36.6 ± 21.8% vs control; *p* = 0.0311) and AT100 epitopes (32.2 ± 17.7% vs control; *p* = 0.031), with a tendency toward a decrease at the AT180 epitope (35.3 ± 23.7% vs control; *p* = 0.056); but not at the PHF1 (101.5 ± 18.3% vs control; *p* = 0.471). Total Tau with Tau5 was unchanged in aged littermates (88.7 ± 44.1% vs control; *p* = 0.408; **Figure 6D**).

## DISCUSSION

The findings in this study demonstrate several novel benefits of CDK5 RNAi treatment. The ameliorative effects of the treatment using an AAV delivery system persist for at least a year after a single dose. Treatment can prevent the Tau pathology and the decline in memory when given before symptoms occur. Also, treatment reduced Tau pathology and induced a clear functional recovery in affected old animals as measured by MWM testing. Those results are consistent with reports of long-term efficacy in mice (Boye et al., 2011), nonhuman primates (Bankiewicz et al., 2006) and in humans (Palfi et al., 2014).

The improvement in the 3xTg-AD mice both in terms of their Tau pathology and their behavior, reinforces and extends several reports on the beneficial effects of clearing hyperphosphorylated Tau (Alvarez et al., 1999; Zheng et al., 2002, 2005, 2010; Wang et al., 2003; Zhang et al., 2004, 2013; Wen et al., 2007; Piedrahita et al., 2010; Castro-Alvarez et al., 2011; Crews et al., 2011). Also, a great deal of evidence supports the occurrence of behavioral deficits in 3xTg-AD mice (Oddo et al., 2003; Billings et al., 2005; Clinton et al., 2007; Gulinello et al., 2009). We showed that aged 3xTg-AD mice easily found the visual platform, and therefore, the animals can learn the task. However, they failed to reach the hidden platform and failed in the probe trial. CDK5 knockdown induced a functional recovery in aged 3xTg-AD at 1 year (long-term) and 3 weeks (short-term) following delivery of the short hairpin. These behavioral improvements were supported by a significant reduction of sarkosyl-insoluble Tau. Hyperphosphorylated Tau decreased in both treatment time-lines, in contrast to CDK5 activity and p25 that decreased in the short-term treatment arm (Piedrahita et al., 2010), suggesting some loss in CDK5 knockdown efficiency over the long term in old 3xTgAD and control mice. Although, this long term silencing maybe prevented the tau hyperphosphorylation in the animals before of the disease began in the hippocampus, in spite of the damage in surrounding brain areas continued, and those are connected with the hippocampus. Also, other altered kinases are involved in different phosphorylation sites of tau in AD (Augustinack et al., 2002), affecting the reduction of CDK5 activity by shCDk5miR in the hippocampus of 3xTgAD mice. In addition, in long term treated old control mice the main prevention of phosphorylation of tau was on ser202, thr205 (AT-8), which has been related with aging or PHF in PS1 KI mice (Kurt et al., 2003). Oppositely, CDK5 down-regulation was not enough to reverse all the phosphorylation sites on tau in old 3xTgAD mice at 3 weeks post-injection.

Tauopathy reduction linked to kinase inhibition has shown behavioral recovery in different animals models. 3xTgAD and transgenic p25 mice treated with diaminothiazoles can reduce tauopathy and improve cognitive function by both CDK5 and GSK3β inhibition (Zhang et al., 2013). These observations are indirectly supported by the demonstration that the reduction of p35 to p25 cleavage by calpain inhibition prevented cognitive alterations in transgenic mouse models (Liang et al., 2010; Medeiros et al., 2012). Furthermore, Crews et al. (2011) have shown rescue of neurogenesis alterations after pharmacological and genetic inhibition of CDK5 in an APP transgenic mice.

On the other side, in our study CDK5 knockdown did not affect old control mice at either long or short-term time points. Main effect of inhibition or knock down of CDK5 on noninjured mice improves relearning skill more than learning process (Hawasli et al., 2007). Reversal learning has different electrophysiological implications and has been linked to the function of the NR2B subunit of the NMDA receptor and AMPA receptor (Dong et al., 2013). However, transfer test was not evaluated in the present study. But could suggest that reducing CDK5 activity can have positive effects on learning and memory independent of its Tau effects, but in younger mice, as shown by Plattner et al. (2014).

The search for therapeutic approaches to prevent or slow the progression of AD is a very high priority. The tauopathy of AD has been a particularly stubborn problem with few, if any, pharmaceutical options. One obstacle to correcting the tauopathy is the paucity of critical targets, which can serve as the basis for future drug discovery. In conclusion, CDK5 knockdown as a treatment and prevention appears to be a reasonable target based on taupathology and behavioral effects in 3xTg-AD mice, and suggest to be safety to the long term in both disease and control conditions, presenting the CDK5miR as a potential translation gene therapy. However, the discovery of CDK5 small molecule inhibitors has been challenging. These results should foster a search for deeper insights into the mechanisms of CDK5 in brain homeostatic control, and improved delivery approaches for safe and long-lasting vectors and ultimately systemic administration of small molecules that can specifically and safely reduce CDK5 activity.

## AUTHOR CONTRIBUTIONS

John F. Castro-Alvarez, design and acquisition data, analysis and interpretation data, manuscript preparation; S. Alejandro Uribe-Arias, acquisition data, analysis and interpretation data; Kenneth S. Kosik, manuscript preparation and critical revision; Gloria P. Cardona-Gómez design, analysis and interpretation data, manuscript preparation and critical revision. All authors read and approved the final manuscript.

## Conflict of Interest Statement

The authors declare that the research was conducted in the absence of any commercial or financial relationships that could be construed as a potential conflict of interest.
